# Structure and semi-sequence-specific RNA binding of Nrd1

**DOI:** 10.1093/nar/gku446

**Published:** 2014-05-23

**Authors:** Veronika Bacikova, Josef Pasulka, Karel Kubicek, Richard Stefl

**Affiliations:** 1 CEITEC—Central European Institute of Technology, Masaryk University, Brno 62500, Czech Republic; 2 National Centre for Biomolecular Research, Faculty of Science, Masaryk University, Brno 62500, Czech Republic

## Abstract

In *Saccharomyces cerevisiae*, the Nrd1-dependent termination and processing pathways play an important role in surveillance and processing of non-coding ribonucleic acids (RNAs). The termination and subsequent processing is dependent on the Nrd1 complex consisting of two RNA-binding proteins Nrd1 and Nab3 and Sen1 helicase. It is established that Nrd1 and Nab3 cooperatively recognize specific termination elements within nascent RNA, GUA[A/G] and UCUU[G], respectively. Interestingly, some transcripts do not require GUA[A/G] motif for transcription termination *in vivo* and binding *in vitro*, suggesting the existence of alternative Nrd1-binding motifs. Here we studied the structure and RNA-binding properties of Nrd1 using nuclear magnetic resonance (NMR), fluorescence anisotropy and phenotypic analyses *in vivo*. We determined the solution structure of a two-domain RNA-binding fragment of Nrd1, formed by an RNA-recognition motif and helix–loop bundle. NMR and fluorescence data show that not only GUA[A/G] but also several other G-rich and AU-rich motifs are able to bind Nrd1 with affinity in a low micromolar range. The broad substrate specificity is achieved by adaptable interaction surfaces of the RNA-recognition motif and helix–loop bundle domains that sandwich the RNA substrates. Our findings have implication for the role of Nrd1 in termination and processing of many non-coding RNAs arising from bidirectional pervasive transcription.

## INTRODUCTION

In yeast, RNA polymerase II (RNAPII) transcribes not only protein coding genes but also a subset of non-coding RNAs (ncRNAs) such as small nuclear (snRNAs), small nucleolar (snoRNAs), micro-RNA precursors, cryptic unstable transcripts (CUTs) and other intergenic and noncoding genes (1). Whereas the transcription of messenger RNA (mRNA) is terminated by a multi-subunit cleavage and polyadenylation complex (1,2), the termination of ncRNA is dependent on the Nrd1 complex (3–5). The latter type of poly(A)-independent transcription termination is linked to subsequent 3′ end processing and RNA degradation by the Trf4-Air2-Mtr4 polyadenylation (TRAMP)/exosome pathway (6,7).

**Figure 1.**
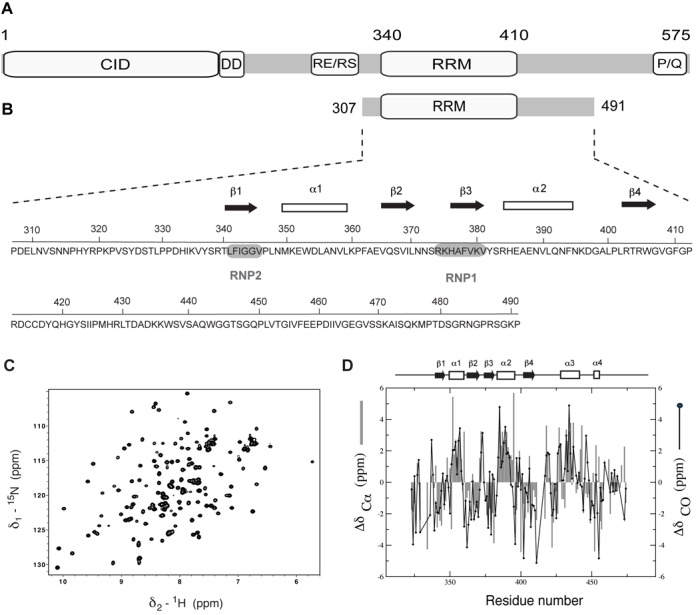
Overview of domain organization of Nrd1, sequence and NMR data of Nrd1_307–491_. (**A**) Scheme of the full-length Nrd1 protein containing CTD-interacting domain (CID), dimerization domain (DD), arginine-glutamate/arginine-serine-rich region (RE/RS), RNA-recognition motif (RRM) and proline-glutamine-rich sequence (P/Q). (**B**) Nrd1_307–491_ construct and its sequence along with highlighted RNP2 and RNP1 sites and predicted secondary structure elements. (**C**) ^1^H-^15^N HSQC spectrum of Nrd1_307–491_ measured at 20°C in 50-mM phosphate buffer (pH = 8) supplemented with 300-mM NaCl and 10-mM β-mercaptoethanol. (**D**) Secondary structure prediction based on Cα and CO chemical shifts correlates with the predicted RRM topology. The plot shows an additional structured region in the C-terminus.

The Nrd1 complex consists of two RNA-binding proteins Nab3 (nuclear polyadenylated RNA-binding 3) and Nrd1 (nuclear pre-mRNA downregulation 1) and the putative helicase Sen1 (3,6,8). Nrd1 is an essential protein and its indispensable role is associated with RNA binding. *NRD1* gene encodes a CTD-interacting domain (CID) and an RNA-recognition motif (RRM) at its N- and C-termini, respectively (Figure 1A and Supplementary Figure S1A). It also contains a dimerization region, allowing the Nrd1-Nab3-heterodimer formation, and a P/Q-rich C-terminal region. Short sequence encoding RE/RS dipeptides suggests a relationship of Nrd1 with metazoan heterogeneous nuclear ribonucleoprotein (hnRNP) family, including also SR (serine/arginine-rich) proteins that function as splicing factors (8). Both Nab3 and Nrd1 proteins recognize specific termination elements within nascent RNA via their RRMs. GUA[A/G] and UCUU[G] are the sequence motifs reported to be recognized by Nrd1 and Nab3, respectively (8–14). Although binding affinities of individual RRM domains of Nrd1 and Nab3 to RNA are in a micromolar range, the Nrd1-Nab3-heterodimer formation results in drastic increase of binding affinity (from micromolar to nanomolar range), due to cooperativity between both proteins (11,12). In addition, Nrd1 CID binds to the C-terminal heptapeptide repetitive sequence (Y_1_-S_2_-P_3_-T_4_-S_5_-P_6_-S_7_) of RNAPII, when phosphorylated at Ser_5_ (15–17). As a consequence of this binding, the Nrd1 complex is recruited in early elongation phase of the transcription cycle when the CTD is highly phosphorylated at Ser_5_. The Nrd1 complex also interacts with the TRAMP/exosome complex and thus mediates subsequent processing/degradation of transcripts (6). The TRAMP complex comprises of poly(A) polymerases Trf4 or Trf5, RNA-binding proteins Air1 or Air2 and the RNA helicase Mtr4. The TRAMP complex targets RNA and adds few subsequent adenines as a signal for degradation by exosome, a complex with 3′ to 5′ exonuclease activity (18–20). Thus, the Nrd1-TRAMP-exosome cooperation plays an irreplaceable role in nuclear RNA surveillance.

The Nrd1-dependent termination pathway was first described for RNAPII transcripts such as snRNAs, snoRNAs (3) and CUTs (4). However, there is increasing evidence of other RNA types, including also RNAs transcribed by RNAPI and III, whose termination and processing can also be dictated by the Nrd1 complex (21–24). The most likely scenario is that incorrect folding of emerging RNA (e.g. due to mutations) exposes the Nrd1- and Nab3-binding sites that are usually hidden in properly folded RNAPI and III transcripts. In general, the availability of single-stranded RNA containing Nrd1- and Nab3-binding sites triggers termination and/or degradation. This assumption is supported by data published in 2011 (25), showing co-transcriptional Nrd1 termination of mRNA. In that interesting experiment, the Nrd1 complex was recruited to emerging mRNA on account of Rho-induced release of RNP proteins, normally protecting RNA sequence. Based on a similar situation when RNA is exposed, the Nrd1 complex can direct premature termination and following degradation of pre-ribosomal, pre-transfer and pre-mRNA as well (21,24,25). On the other hand, the Nrd1 complex does not function only as the surveillance factor during transcription. It acts within 5′ UTR (untranslated region) of NRD1 and IMD2 mRNAs and thereby participates in regulation of protein expression at transcriptional level (3,26). Interestingly, some RNAs can be terminated more than 1 kb downstream from the transcription start site suggesting that non-poly(A) termination is not restricted by CTD-Ser_5_ phosphorylation. For instance, the pre-mRNA of *CTH2* gene is terminated by the Nrd1 pathway around 1.6 kb in order to be post-transcriptionally processed by TRAMP and exosome (27). Next, TLC1 RNA, encoding the template RNA of telomerase, was recently shown to be terminated by the Nrd1 complex close to the mature 3′ poly(A) end (28). Thus, poly(A)-independent termination pathway seems to be a more general mechanism that was originally assumed and recognition of aberrant RNAs as well as termination of non-protein coding transcripts plays a crucial role in maintenance of the equilibrium between transcription and degradation.

Recently, several works dealt with screening of yeast transcriptome to map new possible Nrd1 and Nab3 targets. These data showed that the Nrd1 complex is involved in termination of transcripts of all three RNAPs and confirmed the previously identified sites discovered by genetic and biochemical approaches (10,11). For Nab3, only small variations were observed for Nab3-binding site, such as UCUU, [U]CUUG or GUUCUUGU. For Nrd1, a broader spectrum of targets was observed, varying from the canonical [A/U]GUA[A/G] to other purine-rich motifs including UAAA, AAAU, UGGA or GAAA (13,21–23). In fact, this is not surprising given that GUA[A/G] motif was reported as dispensable for termination *in vivo* (4,11,13). In contrast to this, it was shown that a novel AU-rich sequence motif can enhance the importance of GUAA terminator if present downstream from GUAA. The same work suggests that the efficiency of termination likely depends on the arrangement of termination elements in a ‘supermotif’ (13). This kind of organization would increase variability of terminating sequences and thus make the poly(A)-independent type of termination more general.

Although the recent studies have provided a tremendous amount of data on the function of Nrd1 complex and its importance for transcription termination and processing/degradation, many questions remain, including the central question of how Nrd1 selects a broad range of RNA substrates. Here, we report data from fluorescence anisotropy (FA) measurement in order to describe RNA binding of Nrd1 protein. Surprisingly, our data show that Nrd1 is able to recognize a wide range of RNA motifs, all with affinity in a low micromolar range. The three-dimensional solution structure of RNA-binding fragment of Nrd1 reveals a two-domain architecture composed of a canonical βαββαβ RRM and an extra helix–loop bundle domain. Using NMR titration technique, we analyzed the Nrd1–RNA interactions and found two distinct but partly overlapping RNA-binding regions in the RRM and helix–loop bundle domains for AU-rich and G-rich sequences. These data are supported by the site-specific mutagenesis and the importance of mutated residues is confirmed by FA as well as phenotypic analysis *in vivo*.

## MATERIALS AND METHODS

### Cloning, expression and purification

Deoxyribonucleic acid (DNA) sequence including Nrd1 RRM (307–491) was amplified by polymerase chain reaction and cloned into pET22b plasmid (Novagen) using NdeI and XhoI restriction sites. Resulting construct containing C-terminal His_6_-tag was verified by DNA sequencing and then transformed into *Escherichia coli* BL21-Codon Plus (DE3)-RIPL cells (Stratagene). Bacterial culture was grown at 37°C until OD_600_ ∼ 0.3–0.6 and induced with 1 mM IPTG (isopropyl β-D-thiogalactoside). Protein was overexpressed at 30°C overnight in LB (Luria-Bertani) or minimal M9 medium for FA or NMR measurement, respectively, always supplemented with 50 mg/l of ampicillin. ^15^NH_4_Cl and [U-^13^C_6_]-glucose were added to the M9 medium as a source for isotopic labeling. For expression of protein in highly deuterated background, culture was grown in the M9 medium containing D_2_O (99% atom D) instead of normal water and [U-^13^C_6–_1,2,3,4,5,6,6-D_7_]-glucose (min 99% atom ^13^C, min 97% D). Above that, to prepare protein sample with selectively protonated valine, leucine and isoleucine amino acids in highly deuterated background, ^1^H-^13^C-labeled precursors were added to the M9 medium 1 h before induction. Fifty milligram per liter of ^1^H-^13^C-α-ketobutyrate and 90 mg/l of ^1^H-^13^C-α-ketoisovalerate precursors were sufficient amounts to incorporate into isoleucine and valine/leucine amino acids, respectively (29–31). Cells were harvested by centrifugation and resuspended in lysis buffer (50 mM sodium phosphate, 500 mM sodium chloride, 10 mM beta-mercaptoethanol, protease inhibitors, pH = 8). After disruption of cells the lysate was cleared by centrifugation (21 000 rpm for 1 h) and soluble fraction was loaded on Ni-NTA (nickel-nitrilotriacetic acid) column (Qiagen). The column was washed by lysis buffer containing 10 mM imidazole to wash out non-specifically bound proteins and the Nrd1 protein was eluted by lysis buffer supplemented with a gradient of imidazole (50–500 mM). Elutions with purified protein were dialyzed against dialysis buffer (50 mM sodium phosphate, 300 mM sodium chloride, 10 mM beta-mercaptoethanol, pH = 8). Protein sample was concentrated using Vivaspin 20 (Sartorius) concentrator with 10.000 MW cutoff.

### Generation of Nrd1 mutants

Site-specific Nrd1 mutants were prepared with the QuikChange site-directed mutagenesis kit (Stratagene) and the point mutations were verified by DNA sequence analysis.

### NMR spectroscopy

All NMR experiments were measured on Bruker Avance III systems equipped with cryoprobes of proton frequencies of 600, 700, 900 and 950 MHz at 20°C. The raw data were acquired and processed using Bruker TopSpin 3.0 and analyzed with the use of Sparky 3.113. The resonance assignment of backbone nuclei of Nrd1 RRM was achieved following the standard triple resonance protocol using HNCA, HN(CO)CA, HNCACB and CBCA(CO)NH spectra, further supplemented with deuterated HNCO and HN(CA)CO experiments (32). For the assignment of specifically protonated methyls of isoleucine, leucine and valine, the 4D HCCH methyl NOESY (33), HCCCONH (34) and CCH-TOCSY (35) spectra were used. Titration experiments were done with ^1^H-^15^N-labeled sample where the protein was titrated with aliquots of non-labeled RNA substrate (synthesized by Sigma-Aldrich).

Intermolecular G-quadruplex was prepared as described previously (36). The GCGGGGC RNA sample (0.4 mM) was warmed at 95°C for 5 min and let slowly cool down to room temperature. The formation of G-quadruplex was monitored by ^1^H spectrum. Next, Nrd1 RRM sample was added to the RNA to reach the concentration ratio 1:1 and the 1D spectrum was re-measured. Newly appeared peaks of tryptophan aromatic protons and amide protons in the 10.0 and 9.5 ppm spectral region were compared to those of Nrd1 RRM sample bound to GCGGGGC single-stranded RNA in the ratio of 1:1 after titration experiment.

For the estimation of *R*_2_ and *R*_1_ relaxation parameters, series of 9–11 two-dimensional ^1^H-^15^N spectra were measured in a pseudo-three-dimensional manner on a 700 MHz Bruker Avance III spectrometer equipped with cryoprobe using the pulse schemes as described previously (37). Spectra were processed using TopSpin 3.0 with setting the scaling factor NC_proc to 0. Spectra and peak intensities were then analyzed in Sparky 3.113.

### Structure calculations

The preliminary structure determinations of the free Nrd1 RRM protein were performed with the automated NOE (Nuclear Overhauser Effect) assignment module implemented in the CYANA program (38). This automated NOE assignment procedure is a re-implementation of the former CANDID algorithm (39) on the basis of a probabilistic treatment of the NOE assignment. CYANA carries out automated assignment and distance calibration of NOE intensities, removal of meaningless restraints, structure calculation with torsion angle dynamics and automatic upper distance limit violation analysis. The resultant NOE crosspeak assignments were subsequently confirmed by visual inspection of the spectra. In the next step, CYANA-generated restraints were used for further refinement of the preliminary structures with AMBER 12.0 software suite (40). The ff99SB (41) force field has been used as a modification of the general ff99 (42) for the refinement calculation using a protocol described previously (12,43). From 80 refined structures, the 20 lowest energy conformers were selected to form the final ensemble of structures. Structural quality was assessed using PROCHECK (44) and WHAT IF (45). MOLMOL (46) and PyMOL (http://www.pymol.org) were used for visualization of the Nrd1 molecules.

### FA measurements

The equilibrium binding of Nrd1 to RNA was characterized by fluorescence anisotropy measurement. The RNA was labeled at 5′ end with fluorescein or TAMRA fluorophore. The fluorescein was excited at 488 nm and its emission was collected at 520 nm. For TAMRA fluorophore was set up 561 nm and 581 nm for excitation and emission, respectively. The width of both excitation and emission monochromatic slits was varying from 9 to 14 nm depending on measured RNA sequence. Integration time was set to 3 s. All measurements were conducted on a FluoroMax-4 spectrofluorometer (Horiba Jobin-Yvon). The instrument was equipped with a thermostated cell holder with a Neslab RTE7 water bath (Thermo Scientific). The system was operated by FluorEssence software (version 2.5.3.0 and V3.5, Horiba Jobin-Yvon). All measurements were performed at 20°C in 50 mM sodium phosphate buffer supplemented with 150 mM sodium chloride and 10 mM beta-mercaptoethanol (pH = 8). Ten nanomolar RNA (in a volume of 1.4 ml) was titrated with increasing amounts of Nrd1 protein sample (in 50 mM sodium phosphate buffer containing 300 mM sodium chloride and 10 mM beta-mercaptoethanol, pH = 8). Each data point is an average of three measurements. The data were analyzed using Gnuplot (version 4.4.3) and Xmgrace (version 5.1.16). The data were normalized for visualization purposes and the experimental isotherms were fit to a single-site binding model according to Heyduk and Lee using non-linear least squares regression.

### Yeast growth test analyses

The pRS415 plasmid (CEN, LEU2) containing the *NRD1* gene was used as a template for site-directed mutagenesis (QuikChange site-directed mutagenesis kit, Stratagene). All mutations were verified by DNA sequencing. Wild-type and mutated plasmids were transformed into W303 yeast strain using lithium acetate method. Yeasts were grown in SD-leu-his medium + 2% galactose at 30°C until OD_600_∼1, serially diluted by a factor of 10 in a 96-well plate and dropped on plates with SD-leu-his solid medium supplemented with 2% glucose to repress endogenous *NRD1* gene expression. SD-leu medium containing 2% galactose was used as a positive control. Plates were grown at 30°C and 37°C.

## RESULTS

### Nrd1 RRM requires N- and C-terminal extensions

Nrd1 has two domains, an N-terminal CID and a central RRM (Figure 1A and Supplementary Figure S1A), as identified by Simple Modular Architecture Research Tool (SMART) (47). We determined the structure of Nrd1 CID previously and showed that it is a protein–protein interacting module and it does not bind RNA (17). For structural and RNA-binding studies of *Saccharomyces cerevisiae* Nrd1, we prepared a number of protein constructs (Supplementary Figure S1B). First, based on the secondary structure prediction we designed a set of constructs containing the predicted RRM domain (340–410) with various N- and C-terminal extensions (Supplementary Figure S1B). In *E. coli*, these constructs expressed only insoluble proteins in all tested conditions in which we varied temperatures, IPTG concentrations, vectors, expression cell lines and solubility enhancing tags. Although it was possible to refold the insoluble material after purification under denaturing conditions, the refolded proteins did not give a typical fingerprint of a folded protein in the ^1^H-^15^N HSQC spectra. Hence, we expressed the entire C-terminal part of Nrd1 (307–560), which was soluble, but the quality of ^1^H-^15^N HSQC spectra suffered from a large number of overlapping sharp lines arising from the unstructured C-terminal region of this construct. This construct was subsequently subjected to the limited proteolysis and mass spectrometry, which helped us to identify the domain boundaries for around the RRM of Nrd1. The final construct involves residues from 307 to 491 (Nrd1_307–491_) and it provides a well-dispersed spectrum (Figure 1C and Supplementary Figure S2A). These extra amino acid regions included at both ends of the conserved RRM core domain are crucial for the solubility and proper folding of the recombinant Nrd1 protein in *E. coli*.

### Monomer-dimer equilibrium of Nrd1_307–491_

In our initial NMR experiments, we observed that the ^1^H-^15^N HSQC spectra at different concentrations of Nrd1_307–491_ were not identical (see below). Furthermore, the ^1^H-^15^N HSQC spectra of Nrd1_307–491_ suffered from line width broadening at higher concentrations. Therefore, we set out to investigate whether Nrd1_307–491_ can dimerize or oligomerize with the increasing protein concentration. The backbone resonances of Nrd1_307–491_ (concentration was kept ≤0.4 mM) were assigned using a standard set of double- and triple-resonance experiments (32). The chemical shift deviations of Cα and carbonyl of the assigned backbone resonances of Nrd1_307–491_ from the sequence-dependent random coil values suggest not only the presence of the typical βαββαβ RRM fold but also secondary structure formation in the flanking regions to the RRM (Figure 1D). Knowing the resonance assignments, we performed measurement of longitudinal and transverse relaxation rates, *R*_1_ and *R*_2_, at two different concentrations of Nrd1_307–491_ (0.4 mM and 1.6 mM). The *R*_1_ rates decrease with the increasing size of a molecule, whereas the *R*_2_ rates increase (48), thus these ^15^N relaxation rates can provide qualitative information about the populations of the monomeric and dimeric/oligomeric state of a protein. At the lower concentration (0.4 mM) of Nrd1_307–491_, the average ratio of *R*_2_/*R*_1_ is 30.96 ± 10.88, whereas at the higher concentration (1.6 mM), the average ratio of *R*_2_/*R*_1_ increases to 43.41 ± 16.12, which is an increase of about 50% (Supplementary Figure S3A). The rotational correlation time (*τ*_c_) derived from the whole ^15^N-^1^H T_1_/T_2_ data set for the monomeric state of Nrd1_307–491_ is ∼15 ns. Furthermore, we observed the variation of chemical shifts upon raising the concentration of Nrd1_307–491_ (Supplementary Figure S3B). Altogether, these data are consistent with oligomerization and/or aggregation of Nrd1_307–491_ at higher concentrations.

### Structure of Nrd1_307–491_

To keep the studied ∼21 kDa protein in a monomeric state, we performed all experiments for the NMR titrations, resonance assignments and structural analysis at the protein concentration of ≤0.4 mM. Furthermore, all NMR experiments were measured at a high salt concentration [300-mM NaCl, 50 mM sodium phosphate (pH 8.0) and 10 mM β-mercaptoethanol] to prevent protein precipitation. A combination of two data sets acquired on [^1^H,^13^C,^15^N] and [(70%)^2^H,^13^C,^15^N] samples was used to obtain the backbone resonance assignments (see the Materials and Methods section). With this approach we were able to assign 91% of the backbone resonances in the structured part of the protein (residues 323–456). In addition, several residues in the loops showed no NMR signals. These missing signals are likely a result from the relatively high pH used in the NMR study that was necessary to prevent the precipitation of Nrd1_307–491_. To assign the side-chain resonances, we acquired the 3D HCCH-TOCSY, 3D ^15^N- and ^13^C-separated NOESY-HSQC experiments on the [^1^H,^13^C,^15^N]-labeled sample. Even though these spectra were acquired on a 900-MHz spectrometer equipped with a cryoprobe, they suffered from a low signal-to-noise ratio (Supplementary Figure S2) and did not contain enough information for the side-chain assignments nor the structural determination. Therefore, we adopted a selective protonation strategy via α-ketoisovalerate and α-ketobutyrate precursors to introduce methyl- and ethyl-protonated valines, leucines, and isoleucines into ^2^H, ^13^C, ^15^N-labeled protein (30). The selectively ILV (isoleucine/leucine/valine) protonated sample drastically improved the signal-to-noise ratio in the NOESY spectra (Supplementary Figure S2C). The use of 4D HCCH methyl NOESY (33), HCCCONH (34) and CCH-TOCSY (35) experiments enabled us to assign 96% of the observable methyl and ethyl group resonances.

**Figure 2.**
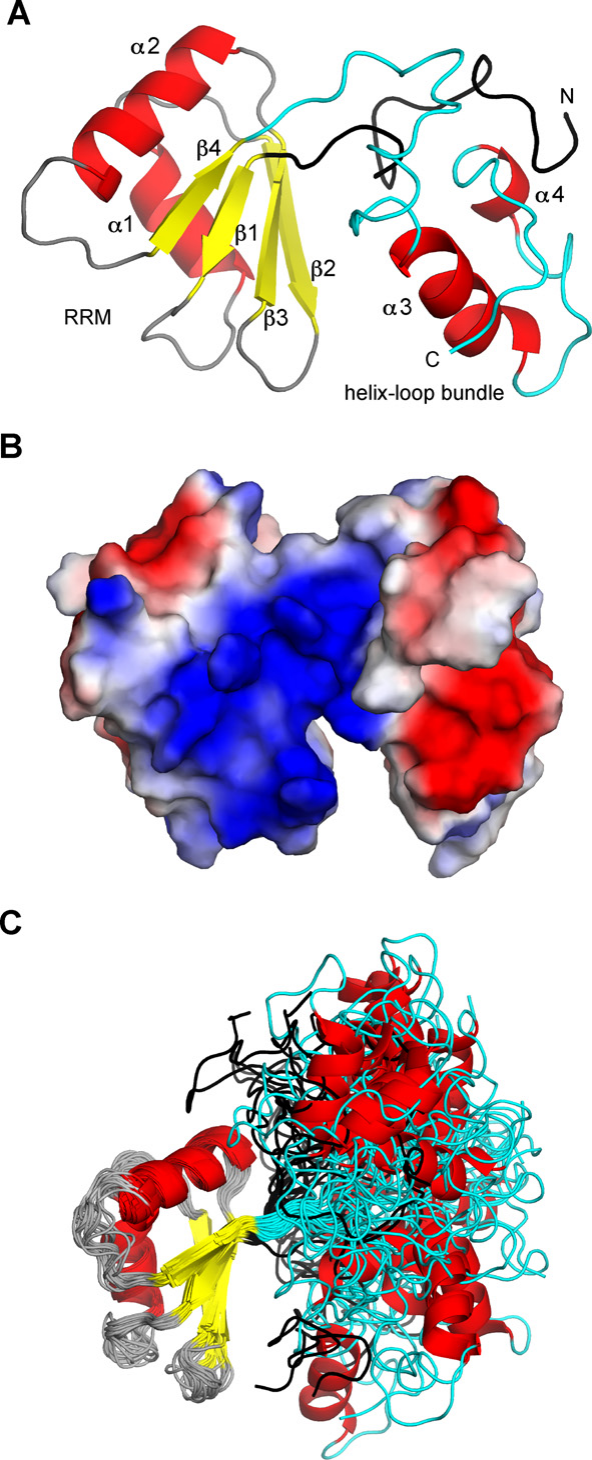
NMR structure of Nrd1_307–491_. (**A**) The lowest energy three-dimensional solution structure of Nrd1_307–491_ consisting of two domains, an RRM with βαββαβ topology and an additional helix–loop bundle domain. The latter domain harbors both N- and C-terminal regions to the RRM of Nrd1 (in black and cyan). The protein is shown as a ribbon model, with β-sheets in yellow and α-helices in red. The structure has been determined using 760 structurally meaningful NOE distance restraints derived from NOESY data acquired on the highly deuterated ^2^H, ^15^N, ^13^C, (Val, Leu, Ile)-methyl, ethyl-protonated protein sample. (**B**) Solvent-accessible surface representation of the representative structure of Nrd1_307–491_ colored by electrostatic potential (blue, positive; red, negative). (**C**) Overlay of the 20 lowest energy structures of the free form of Nrd1_307–491_ over the RRM domain. Figures were generated with PyMOL (Schrödinger, LLC).

Using 760 structurally meaningful NOE distance restraints derived from 3D ^13^C- and ^15^N-edited NOESYs and 4D HCCH NOESY-HSQC (Supplementary Figure S4), we determined the three-dimensional structure of Nrd1_307–491_ by the combined automated NOESY crosspeak assignment (38) and structure calculations with torsion angle dynamics implemented in the program CYANA 2.1 (49), followed by refinement in explicit solvent using AMBER 12 (40). An ensemble of the 20 lowest energy structures along with the best energy structure are shown in Figure 2. A full summary of structural statistics including the backbone ϕ-ψ angle distribution is given in Table 1. The structure is composed of two domains, an RRM (defined by 492 NOEs) and a helix–loop bundle domain (defined by 233 NOEs; Figure 2 and Supplementary Figure S4).

**Table 1.** NMR and refinement statistics for the Nrd1_307–491_

NMR distance and dihedral angle restraints	Nrd1
**Distance restraints**	
Total NOEs	760
Intra-residue	81
Inter-residue	679
Sequential (|*i–j*| = 1)	205
Medium range (1 < |*i-j*| < 5)	148
Long range (|*i–j*| ≥ 5)	326
Hydrogen bond restraints	84
Total NOEs RRM	492
Total NOEs helix-bundle	233
Total NOEs helix bundle + N- and C-term extension	268
**Structure statistics**^a^	
Residual NOE violations (mean ± SD)	
Number > 0.20 Å	0.40 (± 0.60)
Maximum (Å)	0.19 (± 0.04)
**Ramachandran plot statistics**^a,b,c^	
Residues in most favored regions (%)	79.3
Residues in additionally allowed regions (%)	20.1
Residues in generously allowed regions (%)	0.4
Residues in disallowed regions (%)	0.3
Deviations from idealized geometry	
Bond length (Å)	0.00380 ± 0.00008
Bond angles (Å)	1.68 ± 0.01
**Average root mean square deviation to mean structure (Å)**^a^	
RRM domain (340–410)	
Backbone atoms	1.40 ± 0.18
Heavy atoms	2.60 ± 0.30
Helix–loop bundle domain (323–336, 426–456)	
Backbone atoms	2.17 ± 0.82
Heavy atoms	3.71 ± 0.70

^a^Calculated for an ensemble of the 20 lowest energy structures.

^b^Based on PROCHECK analysis.

^c^Calculated for the structured part of the protein construct (323–336, 340–410, 426–456).

The RRM adopts a compact fold with β1α1β2β3α2β4 topology that is similar to the canonical fold of RRM family (50,51). The RRM fold is composed of two α-helices that are packed along a face of a four-stranded antiparallel β-sheet. A central hydrophobic core is composed of conserved residues (Figures 1B and 4D) stabilizing the fold of the domain. Nrd1 RRM contains a well-conserved signature of RRM family, RNP2 and RNP1 sequences (52–54). These two conserved amino acid sequences found between L341-V346 and R374-V381 are located on the β1- and β3-strands, respectively. Their sequence compositions correspond to the general RNP2 and RNP1 consensus [ILV]-[FY]-[ILV]-X-N-L and [RK]-G-[FY]-[GA]-[FY]-[ILV]-X-[FY], respectively, except for the last two amino acids of the RNP2 and for three residues within the RNP1 motif. The presence of aromatic residues in RNP2 and RNP1 sequences, which usually mediates the stacking interaction with RNA bases, along with number of basic and polar residues on the β-sheet surface indicates a potential role of Nrd1 RRM in RNA binding.

**Figure 3.**
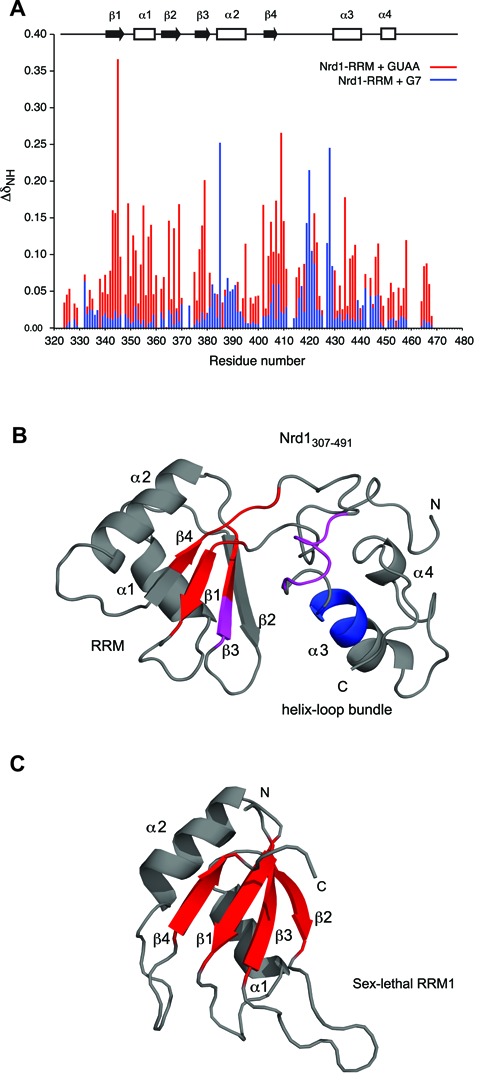
Two types of Nrd1–RNA interaction described by NMR. (**A**) Comparison of Nrd1_307–491_ binding to GUAA (red) and G7 (blue) RNA sequences. GUAA RNA is recognized mostly by residues within β-sheet surface, whereas G7 interaction is mediated by amino acids from the additional helix–loop bundle domain. (**B**) Structure of Nrd1_307–491_ with highlighted regions that are responsible for AU-rich (red) and G-rich (blue) RNA binding. Overlapping region is shown in magenta. RNA-binding surface was colored based on the mutagenesis results. (**C**) A canonical RRM binds RNA via its β-sheet surface (red), exemplified here by the structure of sex-lethal RRM1 [PDB code: 1B7F; (55)].

**Figure 4.**
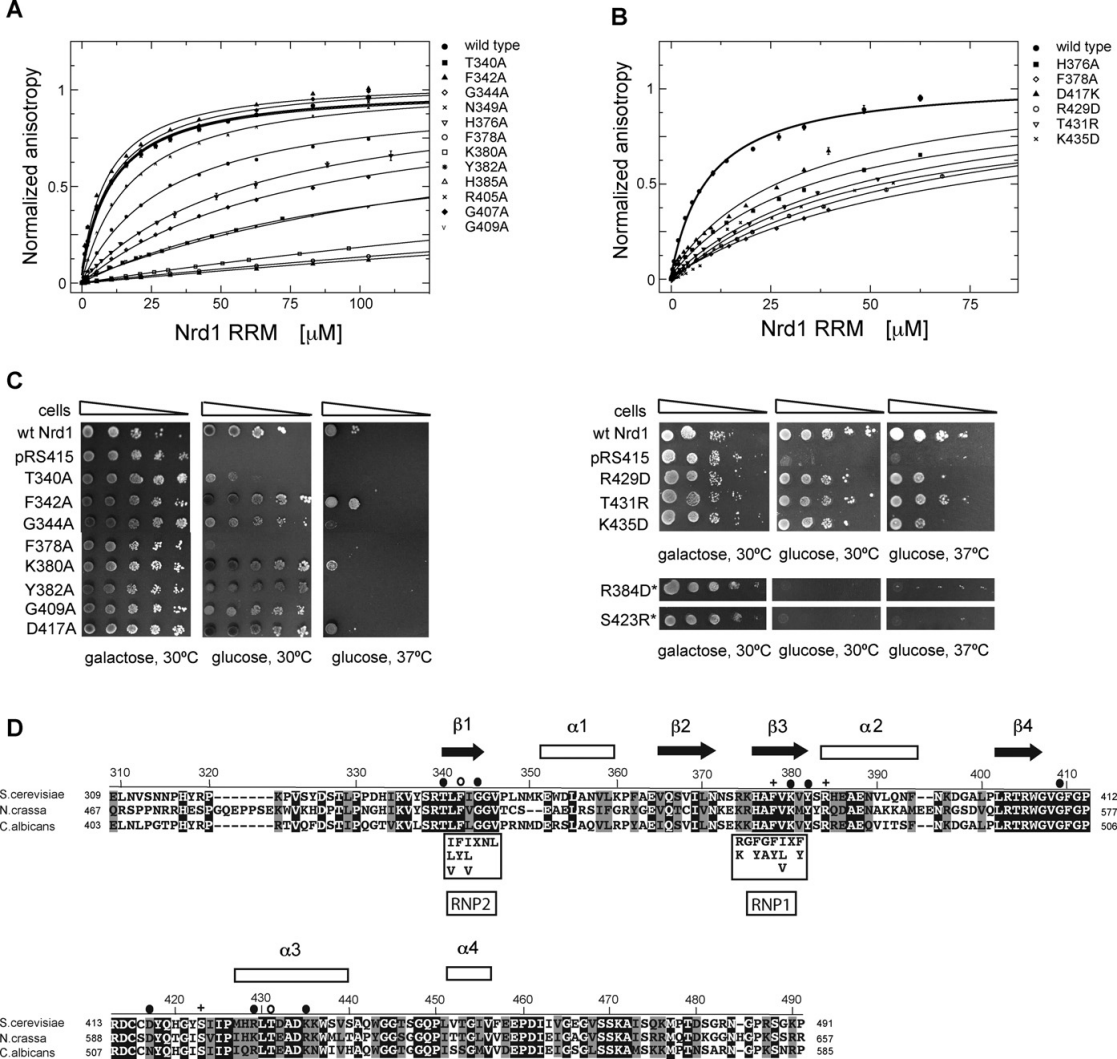
*In vitro* and *in vivo* mutational study of Nrd1. (**A**) GUAA binding by the Nrd1_307–491_ mutants assayed using FA. (**B**) GCGGGGC binding by the Nrd1_307–491_ mutants assayed using FA. (**C**) *In vivo* phenotypic analyses of the Nrd1 mutants. Wt Nrd1 contains non-mutated NRD1 gene, pRS415 is a negative control with empty plasmid without NRD1 gene and the other plasmids contain NRD1 point mutations as denoted. The indicated mutants were expressed episomally from pRS415 plasmids in the yeast strain with the endogenous NRD1 driven by GAL1 promoter. Mutant strains were spotted on plates containing 2% glucose and on a control galactose plate and incubated for 3 days at temperatures indicated. Growth on glucose containing plates leads to the repression of GAL1-driven wild-type Nrd1, and thus shows the functionality of the different Nrd1 mutants. The inviability of Nrd1 variants with asterisks (R384D and S423R) likely results from the insolubility of these mutants; they could not be assayed for RNA binding (see above). (**D**) Alignment of Nrd1_307–491_ from different yeast species along with the secondary structure elements and RNP motifs. Identical residues are highlighted in black, similar ones in gray. The RNP2 and RNP1 consensus sequences are shown in black boxes. Mutated residues with notable phenotype are labeled above the alignment; cross stands for lethality and no RNA binding, filled circle for thermosensitivity and significantly reduced RNA binding, and circle for variants with no defect in the phenotypic analysis but with significantly reduced RNA binding affinity.

The N- and C-terminal regions to the RRM core fold together to form an additional domain composed of two α-helices and loops, called helix–loop bundle domain (Figure 2). The mutual orientation between the RRM and helix–loop bundle domains could not be deduced from the NOESY data, as we found no inter-domain NOEs between the two domains. As a result, the mutual orientation between the two domains is not well defined in the resulted ensemble of calculated structures (Figure 2C). However, the longitudinal relaxation rates (data not shown) are very similar for both domains, indicating a similar flexibility of RRM and helix-bundle domain. The mutual orientation of the two domains is restricted to some extent by the presence of the N-terminal extension that interacts with the helix–loop bundle domain (defined by 35 NOEs) and thus creates a hinge between the two domains. The lowest-energy structure (Figure 2A and B) has a conformation in which the RRM and the helix–loop bundle domain are close to each other, creating a cleft that is highly positively charged (Figure 2B), which indicates a potential site for RNA binding. The absence of inter-domain NOEs could be due to a low number of protons in the selectively ILV-protonated sample of Nrd1_307–491_ or it could reflect higher dynamics at the interface of the two domains.

### Characterization of RNA binding of Nrd1 using FA

Several studies showed that Nrd1 recognizes GUA[A/G] sequence (8–13). To characterize this binding by FA assay we chose the GUAA substrate, as it is more abundant *in vivo*. We determined that Nrd1_307–491_ binds GUAA motif with a *K*_D_ of 10.1 ± 0.8 μM. This is significantly higher affinity compared to UCUU RNA (*K*_D_ > 500 μM) which is the Nab3-binding site and was used as a negative control. Another reported Nrd1 target, UGGA, is bound by Nrd1_307–491_ with *K*_D_ of 94.1 ± 3.9 μM (Supplementary Figure S5A and Table 2). *In vivo*, however, termination sequences are longer and frequently contain several repeats of Nrd1/Nab3-binding sites. Thus, we decided to compare binding of the isolated GUAA motif with binding to win78 RNA. The latter substrate was chosen in accordance with the reported data, that this sequence is sufficient to terminate transcription *in vivo* (13). Win78 RNA contains the Nrd1-binding site GUAA at the 5′ end, two variants of the Nab3-binding site UCUUG and CUUG and an AU-rich sequence. Interestingly, the affinity of Nrd1_307–491_ to win78 is even higher (*K*_D_ = 1.5 ± 0.1 μM) compared to GUAA motif alone, suggesting that longer RNA sequence contributes nonspecifically to the overall binding. We also performed FA measurement with a win78 variant, possessing GUAA mutation to AGCG (win78dNrd1), that is under-represented sequence motif found in *in vivo* SELEX screen for efficient terminators (13). This substrate lacking the Nrd1-binding site had virtually no effect on the binding affinity (Supplementary Figure S5C and Table 2), which corroborates with the observation that the win78 mutant lacking the Nrd1-binding site displayed no termination defects (13). Next, we mutated the Nab3-binding site in addition to the Nrd1 site to avoid any nonspecific interaction, as it is common to find together both Nrd1 and Nab3-binding sites within termination sequences suggesting both sites could contribute to the overall affinity. But, this double-site mutant (win78dNrd1dNab3) did not significantly impair the binding either (a 5-fold drop in affinity). With respect of the recent finding that the AU-rich motif also contributes to efficient termination *in vivo*, we replaced the AU-rich motif in the 3′end of win78 with CACACACA sequence (win78dNrd1dAUrichCA). The win78dNrd1dAUrichCA triple-site mutant displayed similar affinity to the win78 double-site mutant (win78dNrd1dNab3). Next, we replaced all three binding motifs with polyC sequences (win78polyC) that have extremely weak binding affinity to Nrd1_307–491_ (see below). Akin to the previous double- and triple-site mutants, the win78polyC mutant did not significantly impair the binding compared to win78wt (Supplementary Figure S5C and Table 2). Altogether, these data indicate that Nrd1_307–491_ is able to recognize other alternative unknown sequences, likely the AU-rich region bridging the Nrd1- and Nab3-binding motifs in the win78 substrate. In order to map possible RNA targets, we decided to monitor the binding with several short motifs.

**Table 2.** Equilibrium binding of Nrd1_307–491_ to different RNA substrates assayed by fluorescence anisotropy (*K*_D_—dissociation constant)

RNA	*K*_D_ (μM)
GUAA	10.1 ± 0.8
UGGA	94.1 ± 3.9
UCUU	>> 500
UCUUG	41.1 ± 2.7
GGGGGGG	5.7 ± 1.2
UUUUUUU	69.8 ± 3.2
AAAAAAA	32.1 ± 0.9
CCCCCCC	>> 500
AUAUAUA	11.3 ± 0.5
AUUAUUA	14.8 ± 0.4
GUUGUUG	13.0 ± 0.7
UGGUGGU	6.8 ± 0.6
GCGGGCG	13.2 ± 0.5
GCGGGGC	9.8 ± 0.3
CAGCGUC	37.7 ± 1.1
CACACAC	108.5 ± 4.0
win78wt	
GUAAUGAAUUAAGUCUUGAUAUAUAA	1.5 ± 0.1
win78dNrd1	
AGCGUGAAUUAAGUCUUGAUAUAUAA	2.5 ± 0.2
win78dNrd1dNab3	
AGCGUGAAUUAAGAGCGUAUAUAUAA	7.9 ± 0.3
win78dNrd1dAUrichCA	
AGCGUGAAUUAAGUCUUGCACACACA	5.4 ± 0.2
win78polyC	
CCCCUGAAUUAAGCCCCCCCCCCCCC	7.2 ± 0.5

First, we mapped interaction with homoheptamers to see differences between individual nucleotides. In summary, the strength of binding is driven by G>A>U>>C preference, where C_7_ is too weak to be detected (*K*_D_ > 500 μM) (Supplementary Figure S5B and Table 2). As shown in Table 2, cytosine is the only base not recognized by Nrd1. Next, we carried out FA measurements for several AU- and GU-rich sequences (Supplementary Figure S5B and Table 2) as they frequently occur in the win78 terminator. As we expected, Nrd1 binds all these sequences equally well, with a *K*_D_ in the low micromolar range. Lower affinities were observed for termination incompetent motifs (CAGCGUC and CACACAC) that were used to replace the Nrd1- and Nab3-binding motifs in the win78 substrate. Furthermore, the Nab3-binding motif UCUUG is recognized by Nrd1 with a *K*_D_ of 41.1 ± 2.7 μM, which is a comparable affinity to the one of Nab3 RRM–UCUUG interaction (13). Altogether, Nrd1 has a unique feature to interact with a wide range of RNA sequences.

### NMR study of Nrd1–RNA interactions

To investigate the interaction and the binding mode between Nrd1_307–491_ and RNA, we carried out an NMR chemical shift perturbation study with different RNA substrates. First, we titrated Nrd1_307–491_ with GUAA motif. In this RNA titration experiment, we observed that the protein amide resonances changed upon RNA binding from their initial positions, corresponding to the free form, in a stepwise directional manner until they reached their final positions that correspond to the fully bound state, with stoichiometry 1:1 (Supplementary Figure S6). Additional RNA aliquots resulting in excess of RNA induced no further change of chemical shifts, confirming the 1:1 stoichiometry of the complex. These titration data suggest that protein amide resonances are in fast exchange regime between their free and bound forms relative to NMR time-scale. The binding of GUAA to Nrd1_307–491_ induces chemical shift perturbation of the residues shown in Figure 3. These chemical shift changes indicate that the above-mentioned residues are involved in binding to the RNA, or alternatively, could undergo a conformational change upon RNA binding. The chemical shift perturbation profile delineates that the Nrd1–GUAA interaction is mostly mediated through residues in β-strands, especially β1, β3 and β4 (Figure 3A). This is in agreement with the fact that RRM domains usually accommodate RNA on the β-strand surface corresponding to the RNP2 and RNP1 sites (Figure 3C) (55).

As our FA data revealed binding of Nrd1 to other RNA sequences, we titrated Nrd1_307–491_ with other short G-rich and AU-rich RNA motifs. Briefly, the titration results suggest there are two distinct binding regions within Nrd1_307–491_, as shown on the example of GUAA and G_7_ binding (Figure 3A). Whereas the AU-rich sequences, including also GUAA motif, are recognized mostly by the β-sheets of the RRM core domain, the G-rich sequences are mostly bound through the residues of helix–loop bundle domain (Figure 3A). However, we can observe an overlapping region for GUAA and G_7_ interaction (from Gln419 to Tyr422). Given this comparison we can speculate that both domains cooperate to accommodate binding of various RNA sequences.

In the course of FA measurement with G_7_ RNA, it was necessary to prolong the time delay between protein aliquot additions to observe a stable anisotropy values. As guanine-rich sequences possess a unique feature to form quadruplexes, it is likely that the longer incubation time was required to disrupt oligomeric structure of RNA substrate and reach binding equilibrium. Therefore, we performed 1D ^1^H NMR experiment to investigate whether Nrd1 is able to disassemble quadruplex structure. In free form, the 1D ^1^H spectrum of GCGGGGC shows peaks of imino protons around 11 ppm, indicating the presence of quadruplex structure (Supplementary Figure S7). Upon titration with Nrd1_307–491_, the imino peaks of GCGGGGC disappeared and the chemical shifts of amide protons of Nrd1_307–491_ were perturbed in the presence of GCGGGGC RNA. Together, these results suggest that Nrd1_307–491_ interacts with GCGGGGC and it is possible that it can disrupt guanine-quadruplexes in RNA by binding to the single-stranded G-rich sequence.

### Mutational analyses

Based on the titration experiments performed using NMR we could map RNA-binding surface of Nrd1_307–491_ and identify amino acids that are responsible for RNA binding. To confirm the importance of identified residues we prepared point mutants in the RRM and helix–loop bundle domains. The impact of these mutations on RNA binding was tested in a quantitative solution binding assay by FA titration experiments. Furthermore, we also carried out phenotypic study with Nrd1 point mutants *in vivo* to assess whether these mutations influence viability of yeast. First, we assayed the effect of protein mutants for the binding with GUAA. For the RRM that contains the AU-rich binding site, the mutations in the conserved residues of RNP2 and RNP1 (F342A, F378A and K380A) completely abolished the binding to GUAA (Figure 4 and Table 3). Other mutants such as G344A, H376A and G409A showed a 6-to-15-fold decrease in binding affinity of that demonstrated by the wild-type protein. In contrast, mutants N349A, H385A and G407A showed only minor decrease in binding affinity compared to the wild-type Nrd1_307–491_ (Figure 4 and Table 3), suggesting that these amino acids do not bind RNA directly via their side chains or are not in a close proximity of the bound RNA. Overall, these results confirm that Nrd1 RRM specifically recognizes GUAA RNA sequence and the interaction is mediated mostly through amino acids in β-sheets β1, β3 and β4, typical feature for canonical RRM domains (Figure 4A).

**Table 3.** Equilibrium binding of Nrd1 wild-type and point-mutants to GUAA and GCGGGGC RNAs together with their effects on yeast viability

	Nrd1 protein	GUAA *K*_D_ (μM)	GCGGGGC *K*_D_ (μM)	Effect on viability
	Wild type	10.1 ± 0.8	9.8 ± 0.3	Wild type
AU-rich site mutants	T340A	153.1 ± 32.8	n.d.	Thermosensitive
	F342A	>500	29.5 ± 4.0	No
	G344A	80.8 ± 2.2		Thermosensitive
	N349A	10.2 ± 0.5		
	H376A	59.1 ± 2.6	37.5 ± 3.7	
	F378A	>500	73.4 ± 10.7	Lethal
	K380A	441.7 ± 24.7	n.d.	Thermosensitive
	Y382A	32.1 ± 1.3		Thermosensitive
	H385A	10.3 ± 1.7		
	R405A	17.3 ± 0.9		
	G407A	13.9 ± 1.7		
	G409A	157.4 ± 7.7	30.3 ± 3.9	Thermosensitive
G-rich site mutants	D417A	17.9 ± 2.0	31.7 ± 6.0	Thermosensitive
	R429D	24.1 ± 1.7	60.1 ± 3.5	
	T431R	n.d.	48.0 ± 2.9	
	K435D	16.2 ± 1.7	50.5 ± 10.8	

*K*_D_: dissociation constant; n.d.: could not be determined because of poor solubility of the mutants; empty box: not examined.

Next, we introduced mutations in the G-rich binding site present in the helix–loop bundle domain. As shown in Table 3, the chosen mutants showed a 3-to-6-fold decrease in binding affinity of that demonstrated by the wild-type protein (*K*_D_ = 9.8 ± 0.3 μM). For some mutants it was not possible to determine *K*_D_ due to their poor solubility (e.g. R384D or S423R, data not shown). Finally, we tested whether both binding sites are independent or somehow cooperate with each other. To this end, we performed several FA experiments with the AU-rich site mutants and G-rich RNA sequence and vice versa. In this experiment, we would expect no impact on the interaction if the two binding sites were independent. However, the affinity of AU-rich site mutants to GCGGGGC RNA and of G-rich site mutants to GUAA RNA moderately decreased (Table 3), indicating that both sites are not fully independent but may influence each other upon RNA binding.

Nrd1 RRM deletion is lethal for yeast viability (9). To further address the significance of individual RNA-interacting residues of Nrd1_307–491_ for the Nrd1 function *in vivo*, various single amino acid mutants were prepared in a yeast expression vector (pRS415) and introduced into a yeast strain in which the endogenous NRD1 promoter was replaced with the GAL1 promoter (5). To test whether the mutated residues were essential for yeast growth, the resulting transformants were spotted onto glucose containing plates. The shift to glucose represses the expression of the GAL1-driven endogenous NRD1 what completely impairs cell viability (Figure 4C). This lethality was rescued by the wild-type Nrd1 (Figure 4C). Mutating the conserved phenylalanine in the RNP1 motif (F378A) caused lethality (Figure 4C and D). Furthermore, the conserved R384 (G-rich binding residue) and S423 (AU- and G-rich binding residue) were found to be essential for cell viability. However, R384D and S423R variants of Nrd1_307–491_ were insoluble in our *in vitro* experiments, suggesting the reason for the lethality observed *in vivo* (Figure 4C). The other tested single-point mutants either in the AU-rich or G-rich binding sites displayed slow growth phenotypes, providing further support for the functional significance of these residues (Figure 4C).

## DISCUSSION

### Structure of RNA-binding domains of Nrd1

We have determined the structure of RNA-binding fragment of Nrd1, which involves an RRM and a helix–loop bundle domain. The latter domain is important for solubility of the RNA-binding fragment of Nrd1, as a number of different constructs of the isolated RRM without the helix–loop bundle domain were insoluble (Supplementary Figure S1). Due to the solubility issues, the studied construct lacks the upstream RE/RS domain (Supplementary Figure S1). The fold of Nrd1 RRM resembles the one of canonical RRM (50,51,56). RRMs often contain additional structural elements in the N- and/or C-terminal regions to the RRM core, such as β-strand, α-helix or loops, which are important for RNA binding (56,57). In the structure of Nrd1_307–491_, the N- and C-terminal regions fold together to form an additional domain that also contributes to the RNA binding. This is a novel structural feature associated with RRM and the additional helix-bundle domain is crucial for RNA binding of G-tracts or guanine-containing sequences. There are many examples of how multiple RRM-containing proteins can tune affinity and specificity for RNA (58), but very few of those in which RRM is connected to another domain from the same protein. The crystal structure of the N-terminal region of the human La protein, consisting of a La motif and an RRM, in complex with U-tracts, displays that both the La motif and the β-sheet of RRM contribute to RNA binding (59,60). The structure of Nrd1_307–491_ provides yet another example of how the versatility of an RRM can be achieved utilizing an additional domain.

### Broad specificity of Nrd1

There are several examples showing that RRMs can bind RNA in a semi-specific manner (56). For example, the RRMs of polypyrimidine-tract binding protein bind pyrimidine tracts but preferential those with CU-rich tracts (61). Similarly, U2AF65 RRM preferentially binds U-tracts but can adapt to recognize any pyrimidine tract (62). The specificity of U2AF65 RRM is tuned by relocation of flexible side chains and water molecules that mediate interaction with RNAs. GU-rich sequences are recognized by Cstf-64 RRM (63,64). For the Cstf-64 RRM, the semi-specificity is achieved to some extent by a highly dynamic interface capable of accommodating all GU-rich sequences and yet to discriminate against non-GU-rich RNAs.

**Figure 5.**
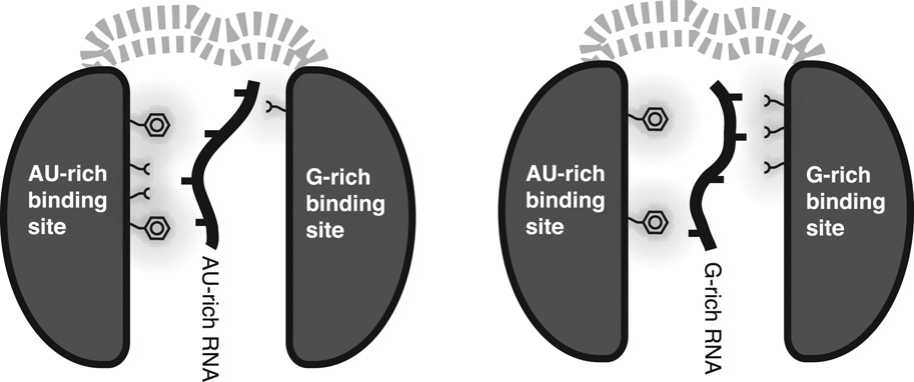
Model of semi-specific binding by Nrd1. The RRM and helix–loop bundle domains of Nrd1 are connected by a two-chain linker and have no fixed mutual orientation. Depending on the sequence, the RNA is primarily accommodated in the AU-rich specific site of the RRM or the G-rich specific sites. It is likely that the mutual arrangement of the domains may change upon RNA binding to accommodate various RNA sequences.

In case of Nrd1, we could not determine the structure of Nrd1_307–491_ bound to either AU-tract or G-tract due to poor behavior and oligomerization or aggregation of the sample at higher concentrations. However, based on the NMR structure of Nrd1_307–491_ in free form (determined at low concentration using fully deuterated protein with selectively protonated ILV residues), NMR titration data with various RNA and binding assays, we propose that the RRM of Nrd1 binds to AU-rich sequences, whereas the helix–loop bundle domain binds to G-rich sequences. Interestingly, both binding sites are not entirely independent of each other as they partly overlap (Figure 3A and B and Supplementary Figure S8A). As the mutual orientation of the two domains is not well defined, it is likely that upon binding to RNA, the domain may differently rearrange to be able to accommodate various sequences (Figure 5). Interestingly, we observed that both Nrd1 RRM residues F378 and F342 of RNP2 and RNP1, respectively, are important for binding to GUAA *in vitro*. However, only the F378A mutant is lethal, whereas the F342A mutant is not. Based on our structural model it is likely that the F378A mutant may be unable to form the ‘closed’ or other RNA-binding competent conformation of the RRM and helix–loop bundle domains, as F378 is located at the interface between the RRM and helix-loop bundle (Figure 2A). Such a structural rationale is also supported by our RNA-binding analysis, which shows that F378 is involved in binding of both AU-rich and G-rich sequences (Figure 3A). It also remains to be seen whether this flexibility between the two domains also exists in the context of the Nrd1-Nab3 heterodimer. For example, the upstream RE/RS domain or another domain from the Nrd1-Nab3 heterodimer could participate in stabilizing the mutual orientation of the two domains or extend the RNA-binding surface, and thus effects the selectivity. As the Nrd1 complex is involved in termination of many non-coding transcripts with a degenerated consensus of terminators (65), we find the hypothesis of mutual rearrangement of the two domains to accommodate various sequences to be plausible.

### Recognition of RNA G-tracts

Recent NMR structures of three quasi-RRMs of hnRNP F bound to G-tract RNA revealed that this special subfamily of RRMs can specifically bind the sequence of three consecutive guanines (66). The recognition is achieved through highly conserved residues located in loops 1, 3, 5, and β-strand 4, instead of the canonical way of binding at the β-sheet of RRM. Although the sequence alignment of RRMs of hnRNP F and Nrd1 shows similarity in loops 1 and 3, these residues of Nrd1 are not perturbed in the NMR titration experiments with G-tracts. Furthermore, we mutated the equivalent residues in loop 1 (L348) and loop 3 (R374 and K375) that are involved in RNA binding of hnRNP F, and these Nrd1 mutants had no impact on G-tract binding (data not shown). Instead, Nrd1 utilizes residues in the helix-bundle domain, such as D417, R429, T431 and K435 to bind G-tracts. Despite the differences in the G-tract recognition mechanisms, both hnRNP F and Nrd1 are able to disrupt the stable quadruplex fold formed by G-tracts. hnRNP F binds to G-tracts in a single-stranded conformation in order to sequester this sequence and prevent formation of guanine quadruplexes or other secondary structure elements (66,67). Such a remodeling of RNA secondary and tertiary structures was shown to be important for the regulation of alternative splicing of the Bcl-x pre-mRNA (66).

### Implication for transcription termination and RNA processing/degradation

Yeast transcriptome-wide analyses derived from *in vivo* cross-linking identified targets for the Nrd1 complex (21,23), corroborating with previously identified sites using genetic and biochemical approaches (10,11). The transcriptome-wide data show only small variations for the Nab3-binding site, such as UCUU, [U]CUUG or GUUCUUGU. In contrast, the Nrd1-binding site is less uniform, varying from [A/U]GUA[A/G] to other purine-rich motifs including UAAA, AAAU, UGGA or GAAA (13,21–23). Furthermore, GUA[A/G] motif is dispensable for sufficient termination *in vivo* (4,11,13). Recent data also revealed that an AU-tract can enhance the importance of GUAA terminator if present downstream from GUAA (13). *In vitro* binding experiments also demonstrated that the mutation of GUAA in the context of artificial CUT (win78) does not affect binding to Nrd1-Nab3 heterodimer (13). Altogether, these data suggest that there may be some alternative Nrd1-binding sites in addition to the canonical GUA[A/G]. Both RNA-binding subunits of the Nrd1 complex, Nrd1 and Nab3, form a heterodimer and cooperate in RNA binding, which not only increases affinity to RNA but also complicates the analysis of sequence specificity of individual subunits. To uncouple the effect of cooperativity, we studied here Nrd1 in isolation and identified that it binds AU-rich, GU-rich and G-rich sequences that nicely corroborate with *in vivo* cross-linking data (21). Furthermore, AU-rich and GU-rich tetranucleotide and pentanucleotide sequences are highly over-represented termination motifs as identified in *in vivo* SELEX (13).

Termination by the Nrd1-dependent pathway is coupled to processing/degradation of transcripts mediated by the TRAMP–exosome complex (4–6). This mechanism leads to full degradation of CUTs and trimming of the sn/snoRNA precursors. It has previously been shown that the Nrd1 complex associates with the exosome/Rrp6p and TRAMP *in vivo* and that the integrity of the Nrd1 complex is required for efficient RNA degradation (6). Given the broad RNA specificity of Nrd1, we suggest that Nrd1 could act as a general RNA-binding subunit of the TRAMP-exosome processing/degradation pathway. Indeed, many CUTs (68) contain no canonical termination motifs of Nrd1 and Nab3, GUA[A/G] and UCUU, respectively, and yet they are processed by the Nrd1/TRAMP/exosome pathway (Supplementary Figure S9). Similarly, Nrd1 pathway serves as a mechanism for transcriptome surveillance, which ensures promoter directionality and prevents transcriptome deregulation (65). Such a transcriptome-wide apparatus that terminates thousands of antisense transcripts selects the desired targets through certain RNA-binding preferences rather than strict specificity for a single motif (65). Future work will be required to elucidate the variations in RNA-binding specificities of Nrd1 and its functional relevance for numerous ncRNAs that are processed or degraded by the Nrd1/TRAMP/exosome pathway.

## ACCESSION NUMBER

The atomic coordinates for the NMR ensemble of the RNA-binding fragment of Nrd1 have been deposited in the Protein Data Bank under accession code 2m88.

## SUPPLEMENTARY DATA

Supplementary Data are available at NAR Online, including [1].

Supplementary Data
