# Supplementary material for: Structure and semi-sequence-specific RNA binding of Nrd1
Source: Nucleic Acids Res. 2014 May 23;42(12):8024–38. doi: 10.1093/nar/gku446 (PMC4081072; doi:10.1093/nar/gku446)
Supplement: Supplementary Data [file supp_42_12_8024__index.html]

Structure and semi-sequence-specific RNA binding of Nrd1 — Supplementary Data 

# Structure and semi-sequence-specific RNA binding of Nrd1

## Supplementary Data

**Files in this Data Supplement:**

- Supplementary Data
