# Supplementary material for: Structure and semi-sequence-specific RNA binding of Nrd1
Source: Nucleic Acids Res. 2014 May 23;42(12):8024–38. doi: 10.1093/nar/gku446 (PMC4081072; doi:10.1093/nar/gku446)
Supplement: Supplementary Data [file supp_gku446_nar-01331-r-2013-File007.doc]

Supplemental Material for

Structure and semi-sequence-specific RNA binding of Nrd1

Veronika Bacikova, Josef Pasulka, Karel Kubicek, and Richard Stefl*

correspondence to: richard.stefl@ceitec.muni.cz (R.S.)

**This file includes:**

Figures: S1 to S9

Reference

**Supplementary Figures**

**
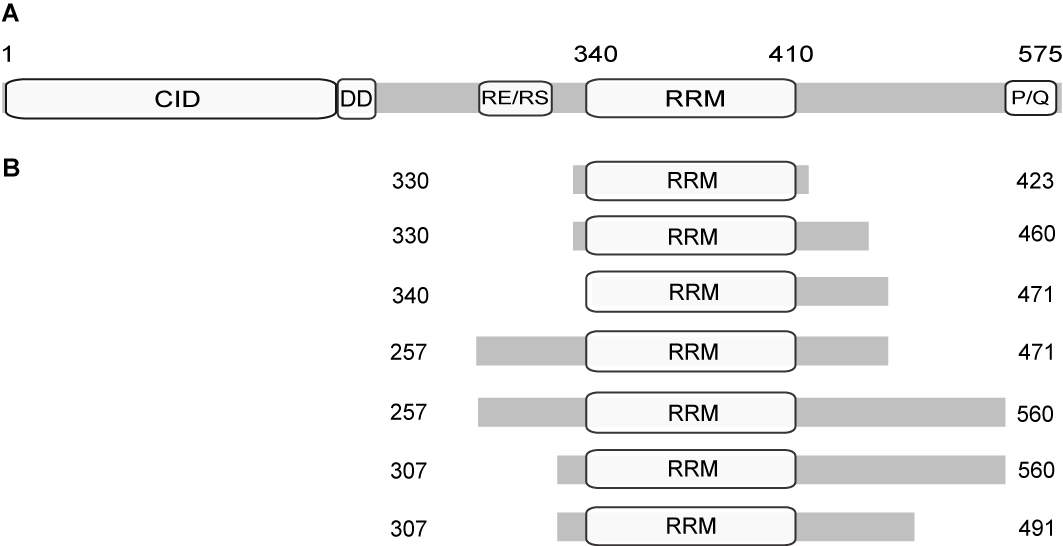
**

**Figure S1.** Overview of domain organization of Nrd1 from *S. cerevisiae* and designed protein constructs. (A) Scheme of the full-length Nrd1 protein containing CTD-interacting domain (CID), dimerization domain (DD), arginine-glutamate/arginine-serine - rich region (RE/RS), RNA-recognition motif (RRM) and proline-glutamine - rich sequence (P/Q). (B) Various Nrd1 fragments used in this study. Only the Nrd1_307-491_ construct was soluble and folded properly during expression in *E. coli*.


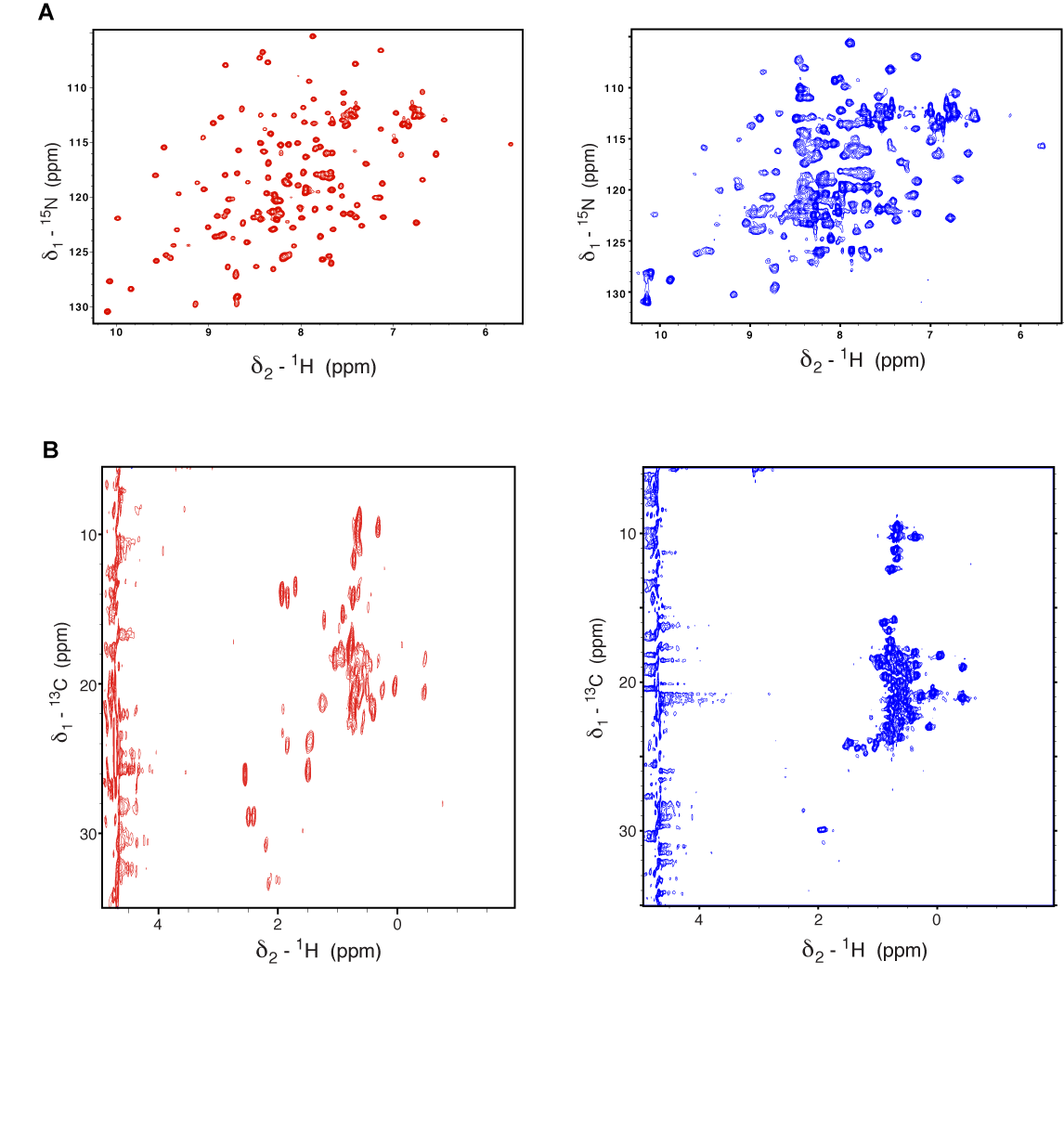


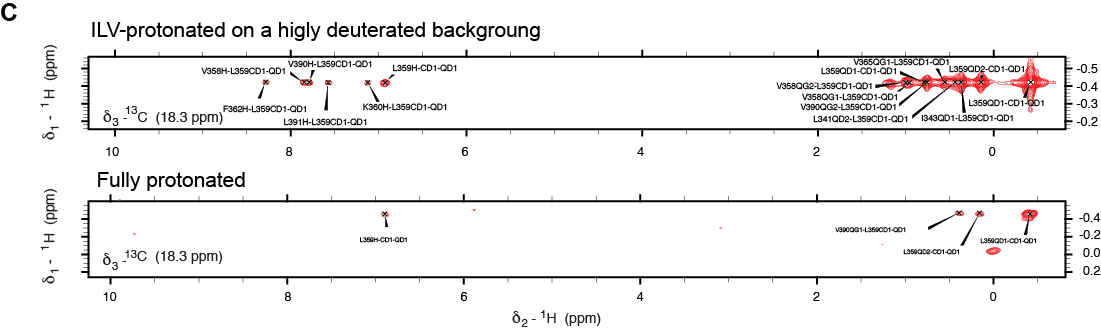


**Figure S2**. A comparison of two-dimensional NMR spectra measured for different Nrd1 samples. (A) ^1^H-^15^N HSQC spectrum of diluted (red) and concentrated (blue) Nrd1_307-491_ sample. (B) ^1^H-^13^C HSQC spectrum (close up at the methyl regions) measured on a 70% deuterated (red) and fully deuterated protein sample with selectively protonated ILV residues (blue). (C) An example of NOESY spectral quality for the fully- and ILV-protonated Nrd1_307-491_ sample, measured at 950 and 900 MHz spectrometers, respectively.


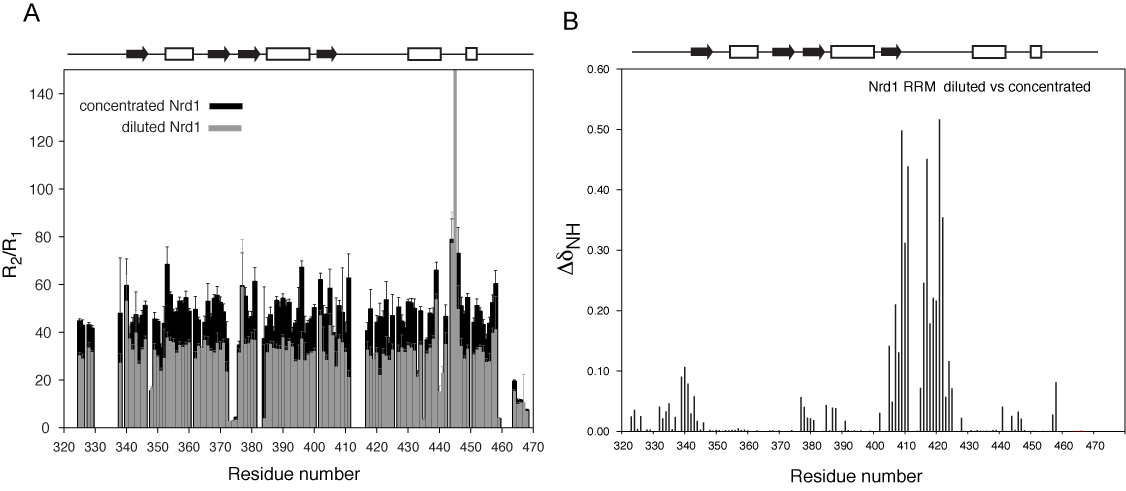


**Figure S3.** The Nrd1_307-491_ protein sample at two different concentrations. (A) Relaxation data of concentrated and diluted Nrd1 sample. The longitudinal and transverse relaxation rates, R_1_ and R_2_, at 0.4 mM and 1.6 mM concentrations of Nrd1_307-491_. (B) A plot of the variations of chemical shifts at two different concentrations (0.4 mM and 1.6 mM) of Nrd1_307-491_.


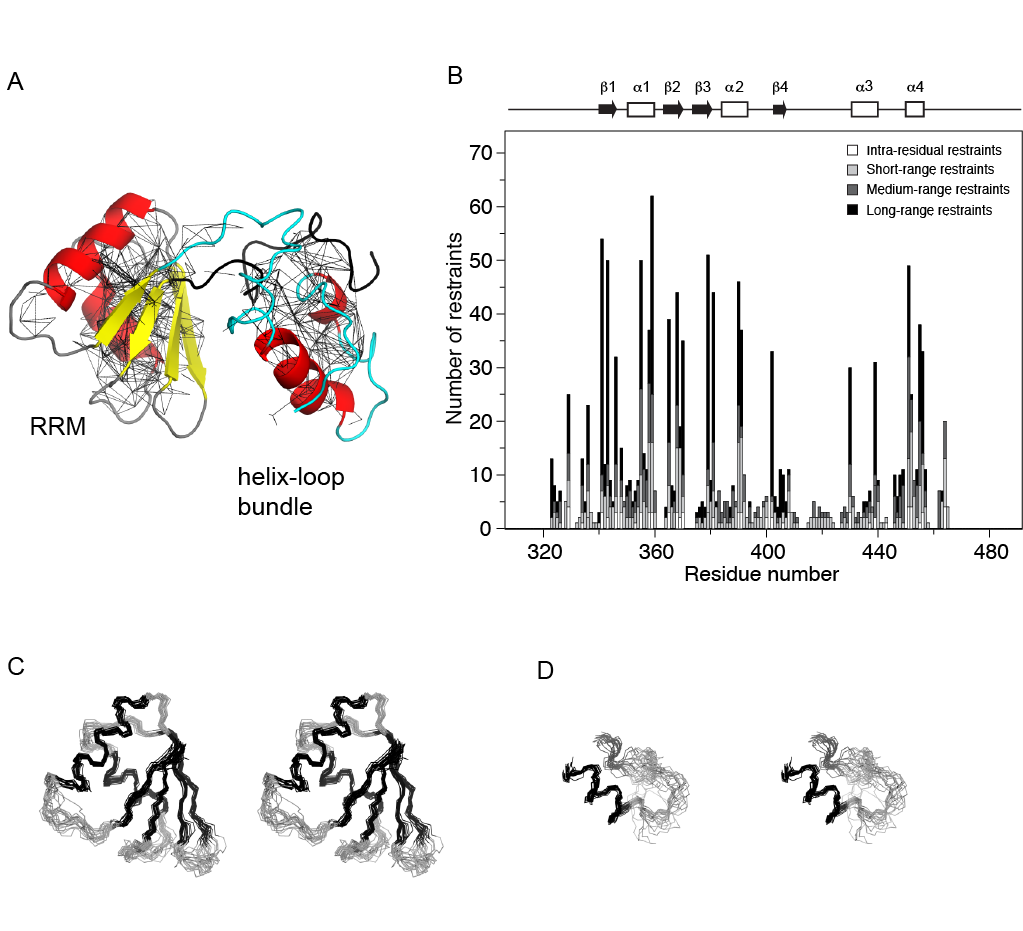


**Figure S4.** (A) The network of distance restraints (black dotted lines) used for the structural calculations is displayed on the three-dimensional structure of Nrd1_307-491_. The distances were derived from the NOESY spectra acquired on the ILV-protonated sample. (B) Distribution of NOE distance restraints by residue. Each column shows intra-residual, short-range (|*i*-*j*|=1), medium-range (1<|*i*-*j*|<5), long-range (|*i*-*j*|≥5). (C) Stereo view overlay of the 20 lowest-energy structures of the RRM domain (residues 340-410) of Nrd1_307-491_. α-helices and β-strands are shown in black and loops are shown in grey. **(**D) Stereo view overlay of the 20 lowest-energy structures of the helix-loop bundle (residues 426-456) of Nrd1_307-491_. α-helices are shown in black and loops are shown in grey.


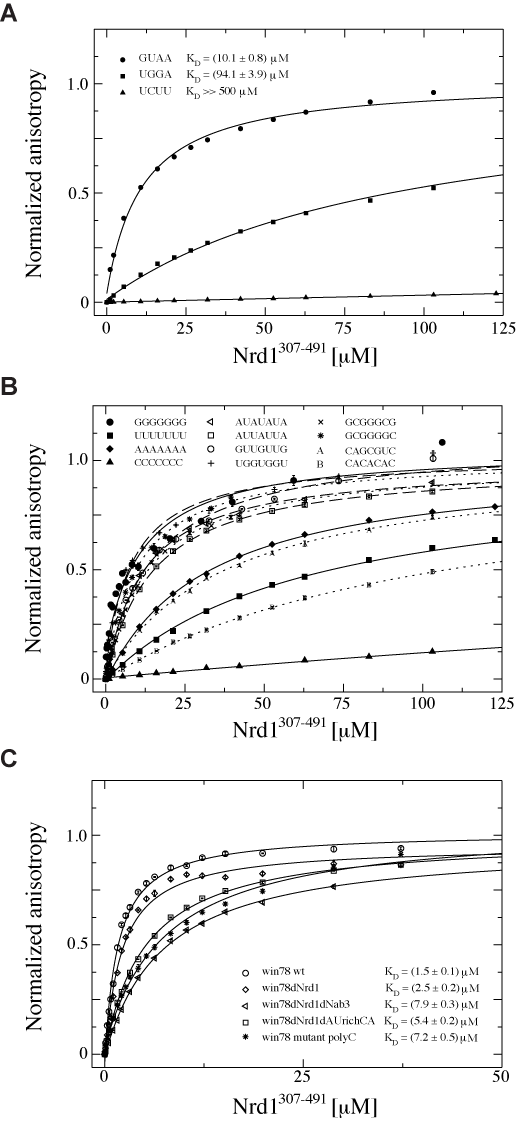


**Figure S5.** RNA binding of Nrd1_307-491_ assayed by fluorescence anisotropy. (A) Binding to GUAA, UGGA and UCUU RNA tetramers. (B) Binding to RNA heptamers, containing just guanines, uracils, adenines and cytosines or their combinations, such as AU-rich, GU-rich, G-rich sequences, CAGCGUC and CACACAC motifs. K_D_ are shown in Table 2. (C) Binding to win78 RNA variants. Win78 wild type (wt) sequence contains one Nrd1, two Nab3-binding sites and an AU-rich region, dNrd1 derivate includes mutation of the Nrd1-binding site, dNrd1dNab3 has mutation of the Nrd1 and one Nab3-binding site, and dNrd1dAUrichCA substrate contains Nrd1-binding site mutation and the AU-rich region replaced by CA-rich sequence.

**
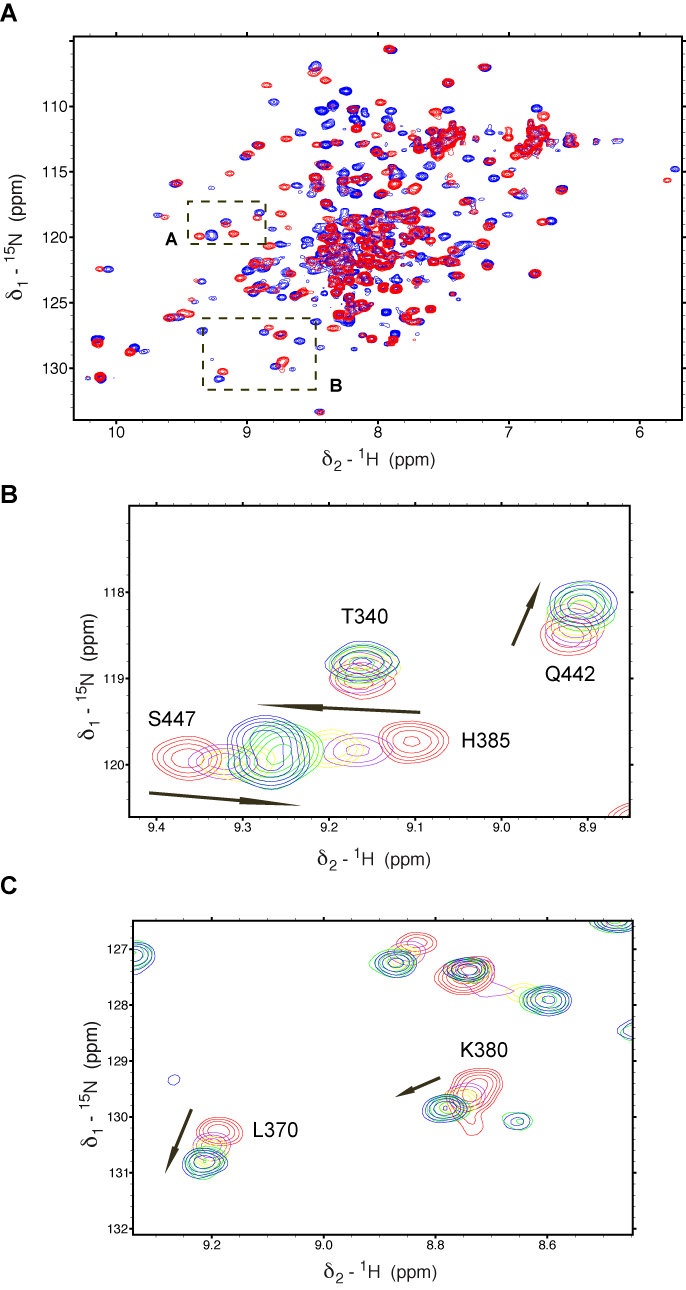
**

**Figure S6.** NMR titration of Nrd1_307-491_ by GUAA RNA. (A) Two-dimensional ^1^H-^15^N HSQC spectra of Nrd1 RRM in free form (red) and bound to GUAA (blue) show changes in chemical shifts of many residues indicating interaction with RNA. (B, C) Close up of some interacting residues; arrows demonstrate direction of individual peak movement during all steps of titration experiment.


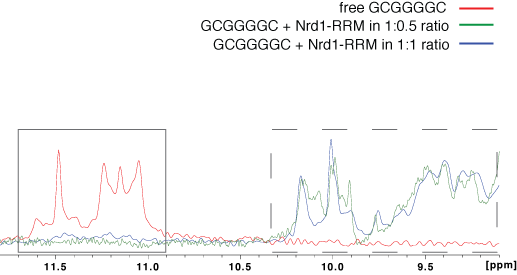


**Figure S7.** ^1^H one-dimensional spectra of Nrd1_307-491_ interacting with GCGGGGC. The figure shows melting of GCGGGGC quadruplex (red spectrum; free RNA) upon interaction with Nrd1_307-491_ in ratio 1:0.5 (green spectrum) monitored by the disappearance of the imino protons of the quadruplex (highlighted in the straight line box). The blue spectrum represents the mixture of GCGGGGC with Nrd1_307-491_ in 1:1 ratio. In the complex, the imino protons of the quadruplex are no more observable and peaks in the spectral region of tryptophane aromatic protons and amide protons (dashed line box) are shifted upon binding of GCGGGGC single stranded RNA with Nrd1_307-491_. All spectra were measured at 293 K on a 600 MHz Bruker Avance III spectrometer equipped with cryoprobe.

**A**

**
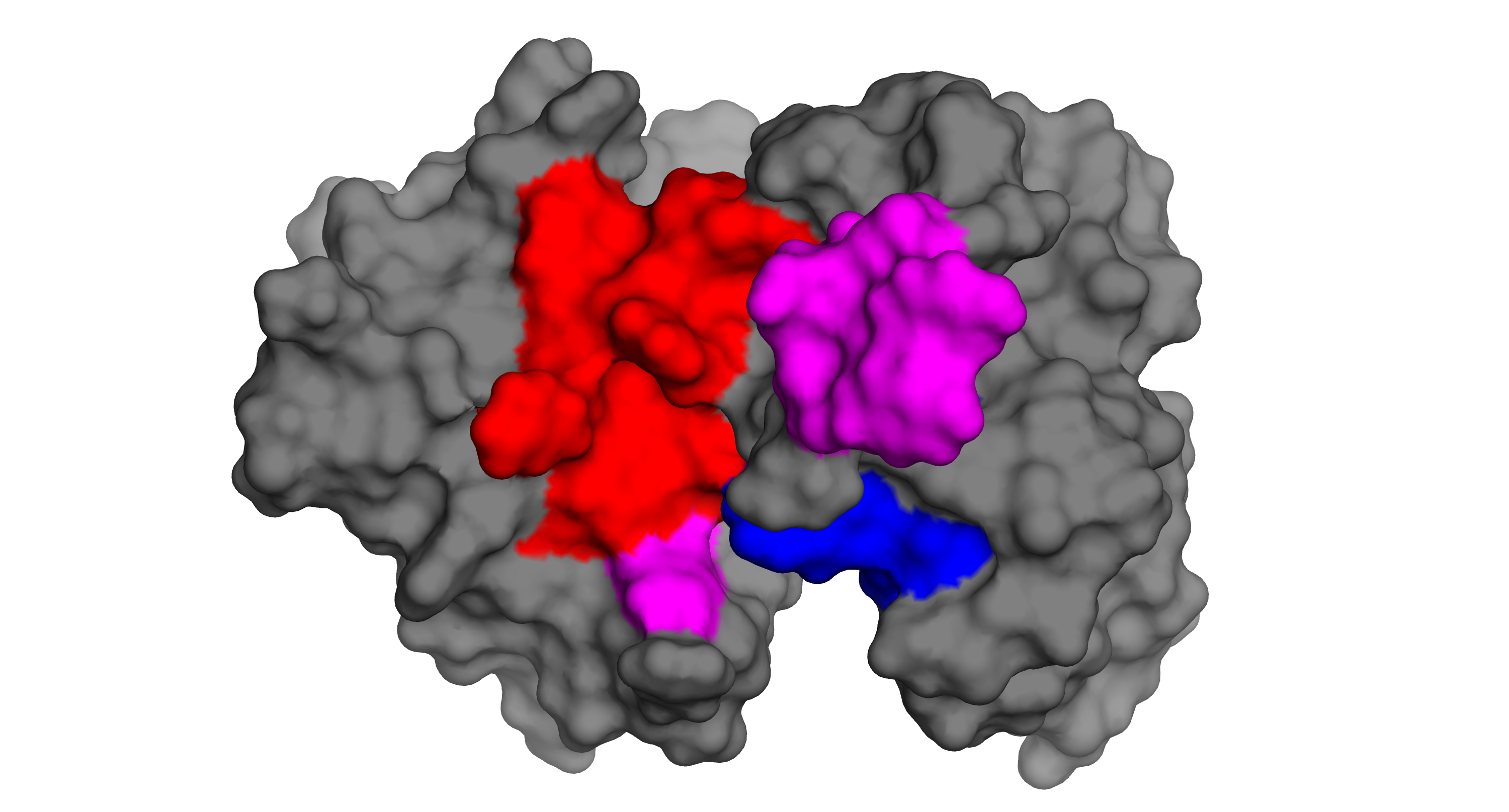
**

**B**

**
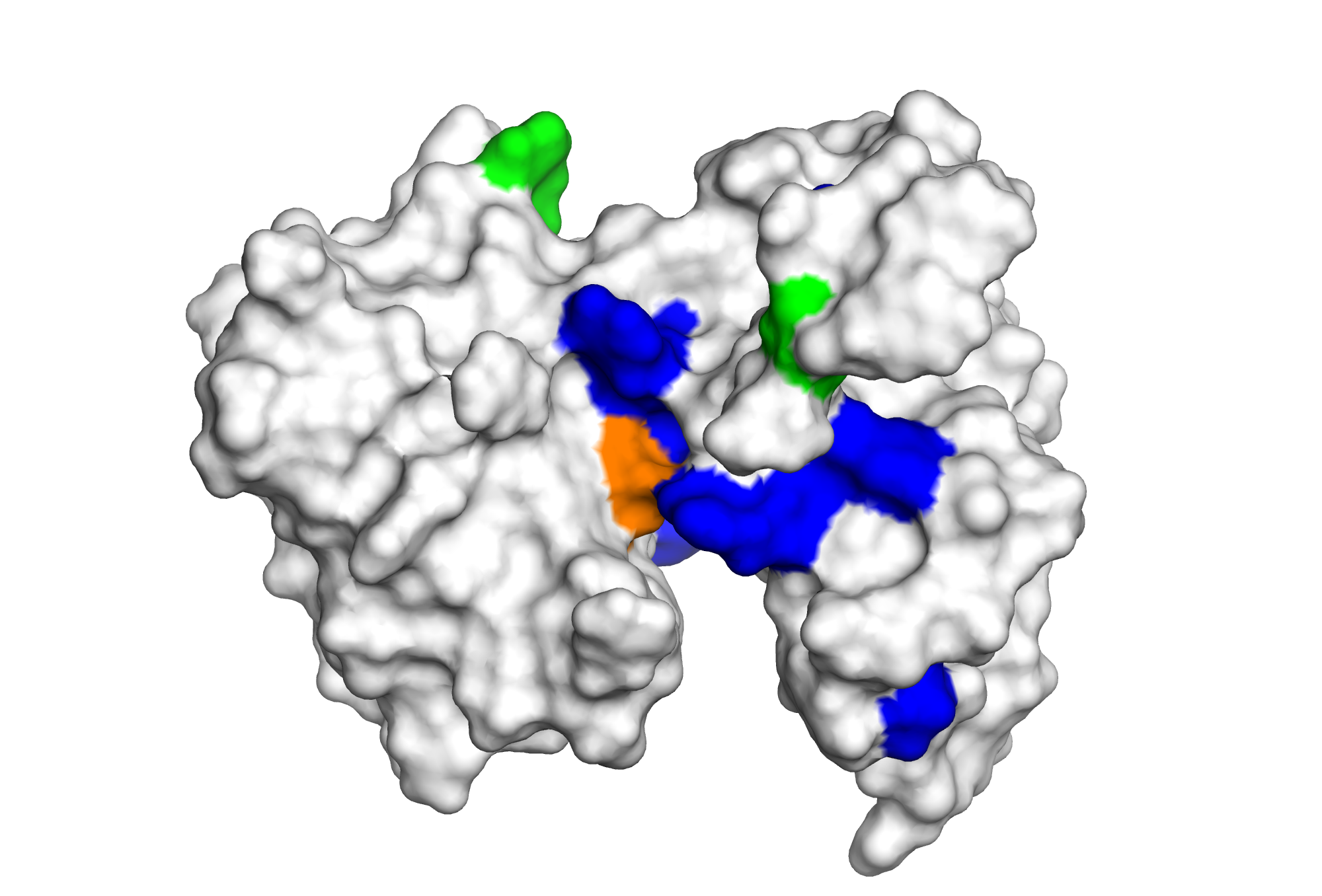
**

**Figure S8.** (A) AU-rich RNA is recognized mostly by residues within β-sheet surface (in red), whereas G-rich interaction is mediated by amino acids from the additional helix-loop bundle domain (in blue). The regions involved in binding of both AU- and G-rich RNAs are shown in magenta. RNA-binding surface was colored based on the largest chemical shift perturbations. (B) Basic patch (R,K, and H residues) of the helix-loop bundle domain is shown in blue. Lethal mutation F378A is shown in orange. The positions of another two lethal mutations R384D and S423R, which resulted in the insoluble Nrd1 variants, are shown in green.


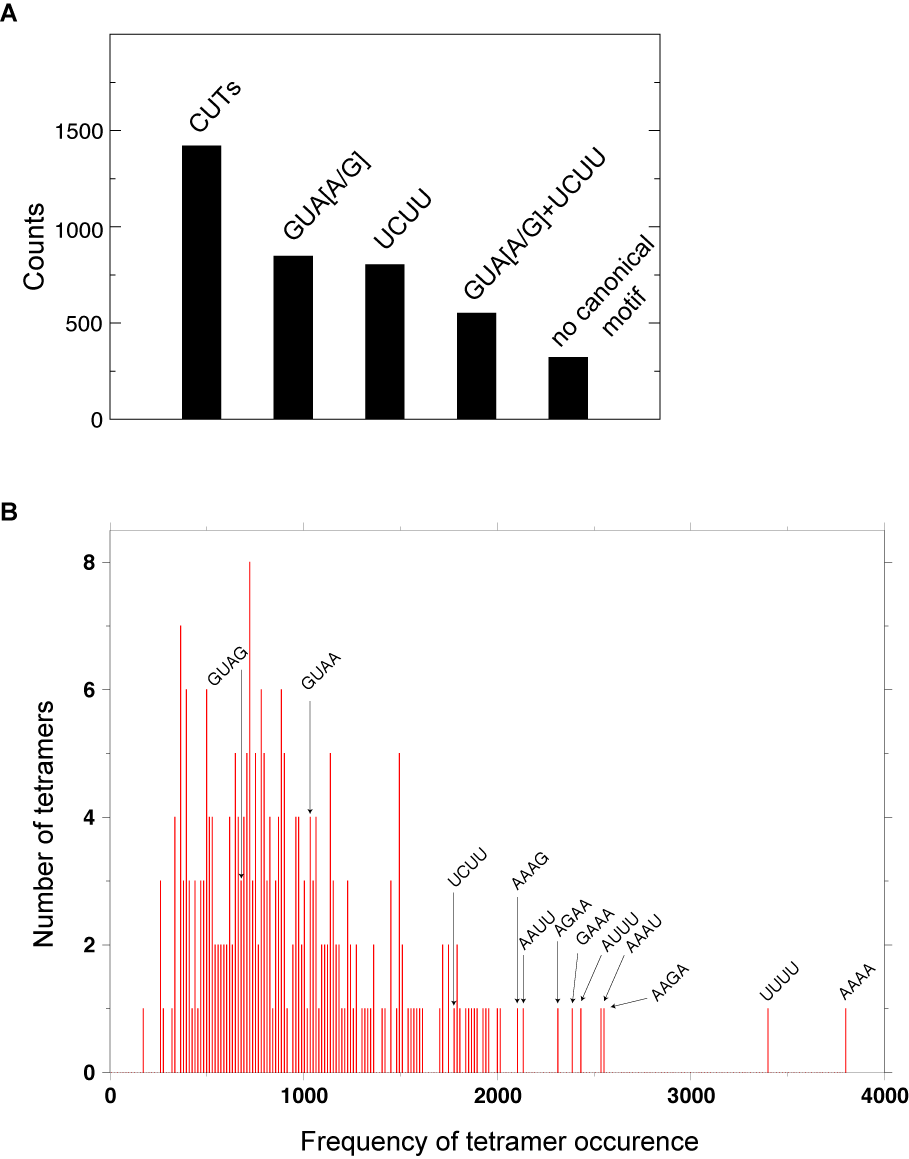


**Figure S9.** Statistical analysis of tetranucleotide motifs present in CUTs. (A) Representation of canonical Nrd1/Nab3-binding sites and other non-canonical motifs within CUTs from *S. cerevisiae* (Neil et al., 2009). (B) Histogram shows tetramer occurrence within CUT sequences; highlighted are the most frequent and canonical tetramer Nrd1/Nab3-binding motifs.

**References**

Neil H, Malabat C, d'Aubenton-Carafa Y, Xu Z, Steinmetz LM, Jacquier A. (2009) Widespread bidirectional promoters are the major source of cryptic transcripts in yeast. *Nature* **457**, 1038-1042.
